# Basic Fibroblast Growth Factor Predicts Cardiovascular Disease Occurrence in Participants from the Veterans Affairs Diabetes Trial

**DOI:** 10.3389/fendo.2013.00183

**Published:** 2013-11-22

**Authors:** Mark B. Zimering, Robert J. Anderson, Ling Ge, Thomas E. Moritz, William C. Duckworth, Investigators for the VADT

**Affiliations:** ^1^Medical Service, Department of Veterans Affairs New Jersey Health Care System, Lyons, NJ, USA; ^2^Rutgers-Robert Wood Johnson Medical School, New Brunswick, NJ, USA; ^3^Hines Cooperative Studies Program Coordinating Center, Veterans Affairs Hospital, Hines, IL, USA; ^4^Division of Epidemiology and Biostatistics, School of Public Health, University of Illinois at Chicago, Chicago, IL, USA; ^5^Phoenix Veterans Affairs Healthcare System, Phoenix, AZ, USA

**Keywords:** basic fibroblast growth factor, type 2 diabetes mellitus, cardiovascular disease

## Abstract

**Aim:** Cardiovascular disease (CVD) is a leading cause of morbidity and mortality in adults with type 2 diabetes mellitus. The aim of the present study was to test whether plasma basic fibroblast growth factor (bFGF) levels predict future CVD occurrence in adults from the Veterans Affairs Diabetes Trial (VADT).

**Methods:** Nearly 400 veterans, 40 years of age or older having a mean baseline diabetes duration of 11.4 years were recruited from outpatient clinics at six geographically distributed sites in the VADT. Within the VADT, they were randomly assigned to intensive or standard glycemic treatment, with follow-up as much as seven and one-half years. CVD occurrence was examined at baseline in the patient population and during randomized treatment. Plasma bFGF was determined with a sensitive, specific two-site enzyme-linked immunoassay at the baseline study visit in all 399 subjects and repeated at the year 1 study visit in a randomly selected subset of 215 subjects.

**Results:** One hundred and five first cardiovascular events occurred in these 399 subjects. The best fit model of risk factors associated with the time to first CVD occurrence (in the study) over a seven and one-half year period had as significant predictors: prior cardiovascular event [hazard ratio (HR) 3.378; 95% confidence intervals (CI) 3.079–3.807; *P* < 0.0001), baseline plasma bFGF (HR 1.008; 95% CI 1.002–1.014; *P* = 0.01), age (HR 1.027; 95% CI 1.004–1.051; *P* = 0.019), baseline plasma triglycerides (HR 1.001; 95% CI 1.000–1.002; *P* = 0.02), and diabetes duration-treatment interaction (*P* = 0.03). Intensive glucose-lowering was associated with significantly decreased hazard ratios for CVD occurrence (0.38–0.63) in patients with known diabetes duration of 0–10 years, and non-significantly increased hazard ratios for CVD occurrence (0.82–1.78) in patients with longer diabetes duration.

**Conclusion:** High level of plasma bFGF is a predictive biomarker of future CVD occurrence in this population of adult type 2 diabetes.

## Introduction

Intensive diabetes treatment slows the development of retinopathy, nephropathy, and neuropathy (1); however, its role in reducing cardiovascular disease (CVD) events in adult type 2 diabetes is more complex. Evidence from the United Kingdom Prospective Diabetes Study (UKPDS) follow-up study indicated a “delayed” beneficial cardiovascular effect from intensive glucose-lowering in newly diagnosed type 2 diabetes (2). Among more advanced type 2 diabetic subjects from the ACCORD, ADVANCE, and veterans affairs diabetes trial (VADT), however, intensive glucose-lowering either did not show a beneficial effect on lowering the rate of cardiovascular events (3) or it increased cardiovascular mortality (i.e., in ACCORD) (4).

Biomarkers that can predict an increased risk for CVD occurrence in a category of adult diabetic patients undergoing substantial treatment intensification would be valuable for guiding patient regarding treatment selection. Basic fibroblast growth factor (bFGF) is a potent angiogenic and smooth muscle cell mitogen which increases in subsets of advanced adult type 2 diabetes having micro-(albuminuria) (5) and/or abdominal obesity (i.e., increased waist/hip ratio) (6). In a planned secondary analysis to the VADT (*n* = 399), we reported that baseline plasma bFGF was a novel significant predictor of the time to first post-baseline occurrence of coronary heart disease (CHD) (7). The aims of the present analysis were (1) to test whether baseline plasma bFGF predicts the time to first post-baseline CVD occurrence in risk models that adjust for treatment-duration interaction, (2) to determine whether elevated bFGF at year 1 of treatment predicts the subsequent occurrence of CVD events in the VADT subjects, and (3) to investigate novel mechanisms for vascular cell growth promotion involving bFGF or long-lasting (FGF-like) autoantibodies in diabetic subsets having increased plasma bFGF.

## Subjects and Methods

### Study subjects

The study design of the VADT has been previously reported (8). Patients with renal insufficiency or congestive heart failure were excluded from participation. Baseline insulin use (yes/no), and occurrence of a macrovascular event prior to baseline (yes/no) were stratification variables jointly used when subjects were randomized at each participating VA site. CVD occurrences encompassing ischemic coronary artery disease, congestive heart failure, cerebrovascular disease, and peripheral arterial disease events comprised the pre-specified endpoint of the main VADT study. Informed consent for the Investigational Review Board-approved substudy was obtained from 399 diabetic subjects who had consented to participate in the main VADT at six outpatient sites. As previously described (7), aliquots of EDTA plasma obtained in the morning from fasting subjects were shipped frozen (dry ice) to a central laboratory [Maverick, Boston Veterans Affairs Medical Center (VAMC), Boston, MA, USA] where they were inventoried and stored at −80°C. Archived, coded frozen EDTA plasma from consecutively enrolled patients was shipped to the laboratory of Dr. Zimering [VA New Jersey Health Care System, Lyons, NJ, USA (VANJ)] where basic fibroblast growth factor immunoreactivity (bFGF-IR) assays were performed. All other assays were performed in the Central Laboratory of the VADT (Tufts University, Boston, MA, USA).

The baseline and follow-up (year 3) clinical characteristics in the study subjects are shown in Table 1.

**Table 1:** **Comparison of baseline and follow-up characteristics for Standard and Intensive treatment groups comprising 399 substudy patients**.

Variable	Baseline			Follow-up^a^		
	STD	INT	*P*-value	STD	INT	*P*-value
	(*N* = 200)	(*N* = 199)		(*N* = 200)	(*N* = 199)	
Age (years)	58.6	60.1	0.08			
Diabetes duration (years)	10.3	12.4	0.007			
Baseline insulin	47%	48%	0.80			
Male	97%	96%	0.99			
Prior CV event	38%	37%	0.79			
Hypertension	73%	68%	0.26			
Race
Non-hispanic white	58%	56%	0.73			
African-American	21%	22%	0.79			
Hispanic	17%	19%	0.68			
Current smoking	16%	20%	0.35	11%	13%	0.43
bFGF (pg/mL)	16.0	14.2	0.50	8.1^b^	8.0^b^	0.95^b^
HbA_1_C (%)	9.6	9.4	0.37	8.8	7.2	<0.0001
Systolic bp (mm Hg)	130.3	131.5	0.51	128.2	124.5	0.03
BMI (kg/m^2^)	31.0	31.0	0.97	31.6	32.5	0.11
Weight (lbs)	212.6	213.2	0.88	216.4	224.3	0.06
Waist/hip ratio	0.99	0.99	0.87	1.00	0.99	0.24
Total chol (mg/dL)	183.2	182.6	0.90	163.4	155.7	0.03
LDL chol (mg/dL)	107.1	107.2	0.96	90.4	86.5	0.21
HDL chol (mg/dL)	36.4	37.6	0.25	38.9	40.1	0.32
Triglycerides (mg/dL)	208.7	184.1	0.16	177.8	148.5	0.006
Serum creat (mg/dL)	1.00	0.99	0.67	1.17	1.15	0.73

*Results are means or percentages, and *P*-values are obtained from the comparison of the two treatment groups*.

*^a^ Year 3 or previous non-missing value prior to year 3*.

*^b^ Year 1 annual visit.Chol, cholesterol; creat, creatinine*.

### Patient subgroups

- A convenience sample consisting of a first group of 26 plasma specimens was used to test for a correlation between plasma bFGF-IR and endothelial cell growth activity (Figure 2). The 26 subjects in this subgroup were consecutively enrolled subjects from three study sites.
- A convenience sample consisting of a second group of 27 plasma specimens was used to test for an association between plasma bFGF-IR and growth stimulatory activity in protein A eluates. The 27 subjects were consecutively enrolled from three study sites.

### Medications

Baseline anti-diabetic medications included oral agents and/or insulin, as previously reported (9). Similar classes of anti-diabetic medications were used in patients randomized to the standard or intensive glycemic treatment groups, but at different doses. Substantial proportions of patients were treated with one or more anti-hypertensive medications including angiotensin converting enzyme inhibitors (67%) or angiotensin receptor blockers (7%). Sixty-two percent of patients reported use of a statin at baseline, and 13% of patients reported baseline use of a fibrate medication.

### Study outcomes

As previously reported (7), CVD was adjudicated by an independent Study Endpoints Committee. The subset of CVD outcomes comprising CHD is defined as myocardial infarction, coronary revascularization, inoperable coronary artery disease, or cardiovascular death. Baseline plasma bFGF-IR was determined in (*n* = 399) subjects and at 1 year post-baseline, a bFGF-IR determination was done due to budgetary constraints in a randomly selected subset of 215 subjects. Data on plasma bFGF-IR was obtained without information about study endpoint occurrence. We separately modeled the association of risk factors with time to first post-baseline cardiovascular (CVD) or to first post-baseline CHD in the 399 subjects for whom such post-randomization data was available.

### Laboratory and clinical measures

Routine laboratory measures were determined by standardized direct enzymatic assay methods (9). The analyzed blood pressure (BP) was the median value of three consecutive determinations recorded from subjects that had been seated and resting for 5-min.

### Plasma samples

#### Basic fibroblast growth factor assays

The determination and stability of basic FGF immunoreactivity (bFGF-IR) in plasma has been previously described (7, 10). Plasma bFGF-IR ranged between 0 and 4 pg/mL in healthy male volunteer blood donors, and there was no effect of age on plasma bFGF level (11).

#### Cell culture and growth assays

Bovine pulmonary artery (BPA) endothelial cells (Clonetics Inc., San Diego, CA, USA) were cultured under previously described conditions (12). Growth-promoting activity is expressed as a percentage of the control cell number (after 4 days incubation in the presence of test fractions) for cells grown in EGM/M199 with 10% FCS without added test fractions. Each point represents the mean of 4–6 determinations. The intra- and inter-assay coefficients of variation were 4 and 7% at 1:50 dilution of protein-A-eluted fractions from plasma of three diabetic subjects (*n* = 3 assays in each patient).

#### Protein A affinity chromatography

Protein A chromatography was carried out as previously described (12). Eluate fractions were stored at 0–4°C. All fractions were sterile filtered (Millipore Corp., Bedford, MA, USA; 0.22 μm) before assay for growth-promoting activity.

#### Chemicals

Recombinant human bFGF was from Austral Biologicals Inc. (San Ramona, CA, USA). All other chemicals and reagents were analytical grade.

#### Protein determinations

Protein concentrations were determined by a bicinchoninic acid protein assay kit (Pierce Chemical Co., Rockford, IL, USA).

#### Statistics

The VADT was conducted using the intention-to-treat principle (9). As previously reported (7), baseline insulin use and baseline cardiovascular event-history (the randomization stratification variables) and glycemic treatment group were included as covariates in models when testing for a bFGF effect on CVD occurrence. Cox proportional hazards regression analysis was used to model the association between baseline risk factors and time to first post-baseline CHD, or CVD occurrence. Backward elimination was used to obtain the best fit model using *P* ≤ 0.05 as the cutoff for variable retention in the final model. From our prior work (5), we hypothesized that increased, post-treatment bFGF (determined at year 1) may reflect suboptimal blockade of the renin-angiotensin system which is a known risk factor for occurrence of CVD events (13). Consistent with our prior methodology (14), year 1 bFGF, when used in models of the risk factors associated with time to first post-year 1 CVD occurrence, was dichotomized at the upper limit of normal reported in adult men (4.4 pg/mL) (11).

## Results

### Baseline and follow-up characteristics in the study patients

Our subject group was comprised of men having the following means: age: 59 years; diabetes duration: 11.4 years; hemoglobin A_1_c: 9.5%; BMI: 31 kg/m^2^; and 37% reported a prior macrovascular event at study entry (Table 1). Baseline clinical characteristics were similar in subjects randomized to standard and intensive treatment, with the exception of mean diabetes duration, which was significantly longer (12.4 vs. 10.3 years) for intensively treated patients (Table 1). At the year 3 study visit, intensive treatment was associated with significantly lower mean glycosylated hemoglobin, systolic BP, plasma total cholesterol, and triglyceride concentrations (Table 1). Mean body weight, BMI, plasma HDL cholesterol, and creatinine concentration increased significantly in all subjects after 3 years’ treatment (data not shown), but the mean values did not differ significantly when comparing standard to intensive treatment patients (Table 1).

### Frequency of occurrence of pooled end points

One hundred and five first CVD events occurred in 105 patients during an average of 6 years of VADT study treatment including 57 primary CVD events in the Standard treatment group and 48 primary CVD events in the Intensive treatment group (data not shown).

### Association between plasma bFGF and first post-baseline occurrence of CVD

**Figure 1:**
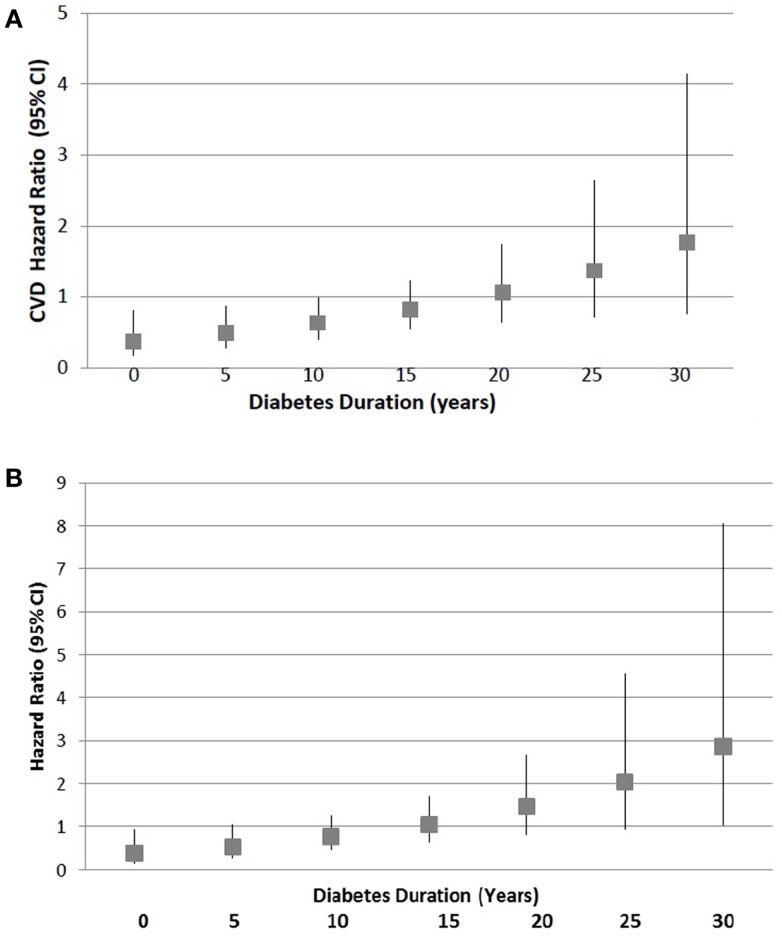
**Hazard ratio for post-baseline (A) CVD or (B) CHD occurrence in intensive treatment group by duration of diabetes known at baseline**. Squares indicate point estimates and bars denote 95% confidence intervals. Point estimates are obtained from the multivariable adjusted model that includes age, prior CV event, baseline bFGF, treatment, duration, and treatment × duration and are illustrated for 5-year intervals between 0–30 years of baseline diabetes duration.

The best-fitting model of risk factors associated with the time to first post-randomization occurrence of the main study CVD endpoint (*n* = 105 events in 399 subjects) had as significant predictors: prior CV event [hazard ratio (HR) 3.378; 95% confidence intervals (CI) 3.079–3.807; *P* < 0.0001], age (HR 1.027; 95% CI 1.004–1.051; *P* = 0.019), baseline bFGF (HR 1.008; 95% CI 1.002–1.014; *P* = 0.013), baseline triglycerides (HR 1.001; 95% CI 1.000–1.002; *P* = 0.015), and duration × treatment interaction (*P* = 0.033) (Table 2). There was no significant association of baseline use of a statin, either ACEi or ARB, or fibrate medication with time to first post-baseline CVD occurrence. There was no significant association of time to first post-baseline CVD occurrence and baseline triglyceride × treatment × duration (*P* = 0.759), baseline triglyceride × treatment (*P* = 0.845) or baseline HDL cholesterol concentration (*P* = 0.441). The (treatment × duration) adjusted model predicted a significantly decreased CVD HR- (CI = 0.38–0.63) associated with intensive glucose-lowering among patients with 10 or fewer years of baseline known diabetes duration, and a non-significant HR (CI = 0.82–1.78) for subjects with a baseline diabetes duration of 15 years or longer (Figure 1).

**Table 2:** **Cox proportional hazard ratio: time to first post-baseline CVD event**.

Variable	HR	95% CI	*P*-value
Baseline bFGF	1.008	1.002–1.014	0.0126
Prior CV event	3.378	3.079–3.807	<0.0001
Age	1.027	1.004–1.051	0.0188
Triglyceride	1.001	1.000–1.002	0.0153
Intensive treatment
5 years diabetes duration	0.489	0.275–0.869	
25 years diabetes duration	1.370	0.712–2.637	

**n* = 399 subjects; HR, hazard ratio; CI, confidence interval*.

### Risk factors associated with ongoing CVD occurrence in advanced diabetes

As previously reported (7), mean plasma bFGF at year 1 decreased significantly compared to baseline levels, but did not differ between STD and INT patients (8.1 vs. 8.0 pg/mL) (Table 1). Of the original 399 study subjects, we were able to obtain repeat plasma bFGF at year 1 in 215 randomly selected patients who did not differ in their mean baseline characteristics from all 399 subjects. Secondary analyses suggests that for patients having the longest baseline duration of diabetes (≥15 years), patient age (HR 1.052; 95% CI 1.006–1.098; *P* = 0.0316) is an important predictor of time to first post-year 1 CVD occurrence (22 events in 71 patients). Excluding age from our model, increased year 1 plasma bFGF (>4 pg/mL) appeared to be a nearly significant predictor (HR 2.439; 95% CI 1.522–3.356; *P* = 0.0518) of time to first post-year 1 CVD occurrence (22 events in 71 patients) among patients with baseline diabetes duration ≥15 years.

### Increased bioactivity in VADT plasma having elevated bFGF

**Figure 2:**
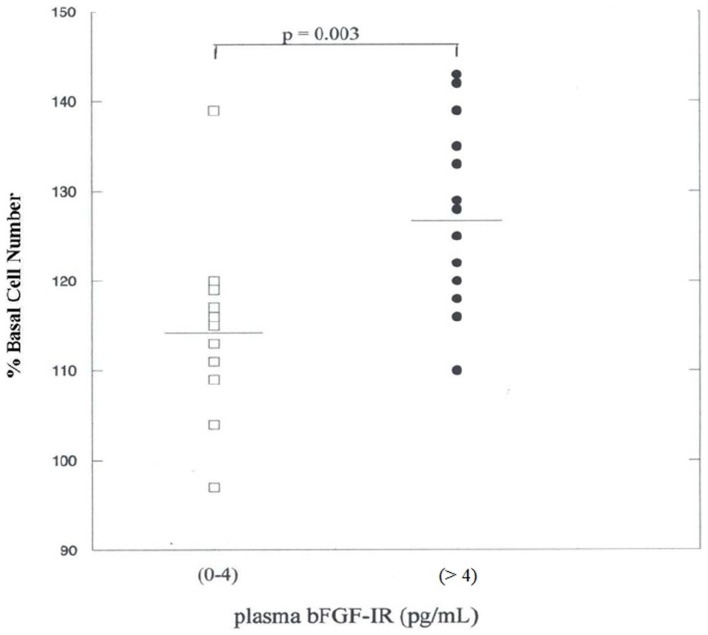
**Endothelial cell bioactivity in the 25–75% ammonium sulfate pellet of plasma**. Ammonium sulfate pellet fractions from plasma in 26 consecutively enrolled VADT subjects were tested for endothelial cell growth promotion after 4 days of incubation as described in Materials and Methods. Each point represents the mean of four–six determinations.

Since basic FGF is a potent endothelial and smooth muscle cell mitogen which may contribute to cell proliferation associated with atherosclerosis, we compared endothelial cell bioactivity in plasma from VADT subjects having low vs. elevated (>4 pg/mL) plasma bFGF. Mean activity in the 25–75% ammonium sulfate pellet fraction of plasma (127 ± 10%) from 14, consecutively enrolled VADT subjects with elevated bFGF-IR (>4 pg/mL) significantly exceeded mean activity (113 ± 10%) in 12 consecutively enrolled VADT subjects with normal bFGF-IR (0–4 pg/mL, *P* = 0.003) (Figure 2).

**Figure 3:**
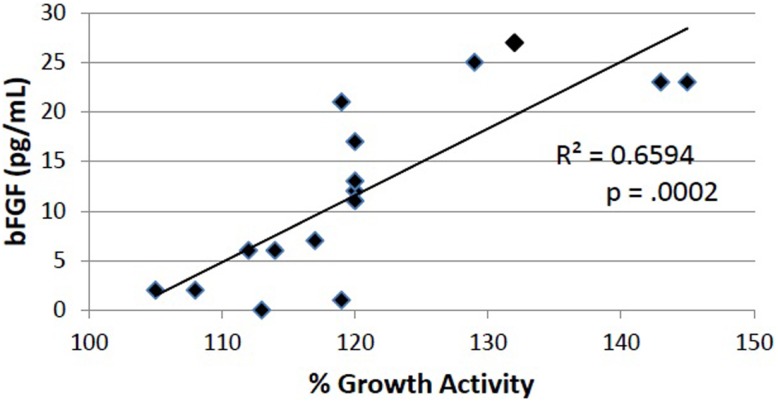
**Endothelial cell stimulatory activity in protein A eluate fractions in 16 VADT plasmas was significantly correlated with increasing plasma bFGF-IR concentration**. Growth activity was determined after 4 days’ incubation as described in Materials and Methods. Each point represents the mean of quadruplicate determinations.

Basic FGF lacks an amino terminal signal sequence (15), and is sequestered in cells (16). Yet in subsets of endocrine tumor patients having elevated plasma bFGF, we have reported the occurrence of long-lasting, highly potent FGF-like autoantibodies (12, 17). In the present study, we examined the protein A eluates from 27 consecutively enrolled VADT subjects having plasma bFGF-IR ranging from 0 to 29 pg/mL for properties resembling autoantibodies. In 16 of 27 subjects, endothelial cell bioactivity in the protein A eluate fraction was stimulatory, and there was a significant correlation (*R* = 0.81; *P* = 0.0002) between plasma bFGF level and the level of growth stimulatory activity in the protein A eluate fraction of plasma (Figure 3).

## Discussion

The current findings suggest that baseline plasma bFGF may be a novel significant predictor of CVD occurrence in a subset of obese, adult male veterans with type 2 diabetes. Following adjustment for a significant treatment − duration interaction, and baseline plasma triglycerides, baseline plasma bFGF was still a significant predictor of the time to first post-baseline CVD occurrence in 399 VADT subjects. This is the first evidence that plasma bFGF is a novel candidate marker of CVD risk in a subset of obese advanced type 2 diabetes.

Plasma basic FGF is low or undetectable in healthy subjects (11), but increases in micro-albuminuric adult type 2 DM (5) or in the presence of coronary artery disease (18). Growth stimulatory activity in the 25–75% ammonium sulfate pellet fraction in VADT plasma having elevated bFGF may be due (in part) to bFGF, or autoantibodies which can bind bFGF or mimic bFGF’s growth activity. In support of the latter two possibilities, our preliminary experiments indicated that even low concentrations (1–2 μg/mL) of protein A eluates from subsets of advanced adult type 1 autoimmune diabetes (*n* = 2) or advanced adult type 2 DM having chronic kidney disease (*n* = 3) could be completely neutralized by co-incubating endothelial cells with specific anti-bFGF antibodies. This result suggests that some of the stimulatory activity in protein A eluates from plasma of advanced diabetes subjects may be FGF-like or due to an IgG which can mimic FGF.

Although the tissue sources of increased plasma bFGF in obese subjects with type 2 diabetes are not known, increased waist-hip ratio was significantly associated with substantially increased plasma bFGF in the VADT substudy (6). Angiotensin II stimulates the release of visceral adipocytokines (TNF-alpha, IL-1, IF-gamma) which then act to induce endothelial cell release of bFGF (19). IF-gamma activates macrophage phagocytic function, and promotes B cell isotype switching to the synthesis of IgG (20). Thus the known pro-immune effects from renin-angiotensin system (RAS) activation may act to alter long-lasting IgG autoantibodies to circulating forms of bFGF in diabetic subsets (e.g., VADT) having substantially increased plasma bFGF. Weight gain and/or salt and water retention frequently accompany glycemic treatment intensification, and the latter has been reported to enhance the expression of angiotensin II receptors in rats (21). The current finding of a significant association between baseline bFGF level and increased CVD risk may reflect underlying role(s) for angiotensin II in promoting vascular hypertrophy, atherosclerosis, and immune interactions involving high plasma bFGF. Our preliminary findings of significant or nearly significant associations between advanced age or increased (>4 pg/mL) year 1 plasma bFGF, respectively and the ongoing risk for CVD occurrence in patients having longer-duration of diabetes (≥15 years) are hypothesis-generating and may warrant further investigation. These results are consistent with known RAS activation in older subjects or in those having year 1 plasma bFGF >4 pg/mL (5). Older patients with type 1 diabetes were more likely to experience adverse hemodynamic responses to RAS activation than their younger counterparts (22).

Increased affinity of the FGF-like plasma autoantibodies in diabetic subjects for hydroxyapatite (our preliminary results) suggests that the autoantibodies may localize to calcified areas in advanced coronary atherosclerotic plaque and also may promote the local cellular proliferation typically found with atherosclerosis. Intensive glycemic control may have been associated with a lower rate of CVD occurrences in our subgroup of recent-onset type 2 diabetes by decreasing the occurrence of microvascular damage leading to the increase of bFGF (23). Findings in the VADT substudy of Reaven et al. (24, 25), namely, that intensive control lowered the subsequent risk of CVD occurrences in subjects having lower baseline coronary calcium, and that albuminuria was a risk factor for progression of coronary calcium, are consistent with the possibility of synergistic interactions between coronary calcium and hydroxyapatite-avid, growth-stimulating autoantibodies.

Atherogenic dyslipidemia has been associated with increased residual cardiovascular risk in obese subjects with type 2 diabetes (26). Consistent with results in the main VADT (9), our substudy group had substantially increased mean baseline plasma triglycerides which remained elevated despite substantial glycemic improvement (Table 1). Our multivariable risk model predicts that each 50 mg/dL increase in baseline plasma triglycerides is associated with a 5% increase in the hazard rate for CVD occurrence. The HR associated with increased bFGF (HR 1.008; 95% CI 1.002–1.014) was only modestly increased, but is likely to be significant for the 20% of VADT subjects who had baseline plasma bFGF >20 pg/mL. Patients having an elevation in plasma bFGF of 20 pg/mL compared to control patients would experience 1.17 times the hazard rate for CVD occurrence. The smaller subgroup of patients (6%) having an elevation in bFGF of 50 pg/mL would experience 1.5 times the hazard rate for CVD occurrence compared to control patients with non-elevated bFGF. Taken together with our prior finding of a significant association between markedly increased plasma bFGF (>20 pg/mL) and increased plasma plasminogen activator inhibitor-1 level (6), the current results suggests effects of bFGF on endothelial cells (i.e., proliferation, elaboration of PAI-1) or smooth muscle cells which may contribute to clinically significant increases in CHD or CVD risk in a subset of obese, advanced type 2 DM.

“Vascular metabolic memory” refers to the association between improved glycemic control in adult type 1 diabetes and a substantially reduced risk for later CVD occurrence (27). Since long-standing poor glycemic control has been significantly associated with increased FGF-like plasma bioactivity in adult type 2 DM (5), vascular injury leading to increased plasma bFGF (23) may be one of the unknown mechanisms underlying an association between prior glycemia and future CVD risk.

A limitation of our study is that the results are based on moderate to small sizes and may only reflect the experience of middle-aged and older obese men with treatment-resistant diabetes. The present findings need to be confirmed in other populations with type 2 diabetes. Our finding of a significant treatment x duration interaction effect on the risk for CVD occurrence in adult type 2 diabetes is consistent with results from the main VADT (28), but needs to be interpreted cautiously since it was based on *post hoc* analysis in an embedded subgroup of the main VADT.

In conclusion, the present findings suggest that baseline plasma bFGF may be a marker of CVD risk in adult male veterans with type 2 diabetes. These results suggest that increased plasma bFGF may drive cell proliferation and be involved in the mechanism for increased CVD occurrence in older adults with advanced type 2 diabetes mellitus.

## Conflict of Interest Statement

The authors declare that the research was conducted in the absence of any commercial or financial relationships that could be construed as a potential conflict of interest.
